# Bisphosphonates can prevent recurrent hip fracture and reduce the mortality in osteoporotic patient with hip fracture: A meta-analysis

**DOI:** 10.12669/pjms.322.9435

**Published:** 2016

**Authors:** Jing Peng, Yong Liu, Long Chen, Kun Peng, Zhao Xu, Dagang Zhang, Zhou Xiang

**Affiliations:** 1 Jing Peng, Department of Orthopedics, West China Hospital, Sichuan University, Chengdu, Sichuan, China; 2 Yong Liu, Department of Orthopedics, Bao Ji Central Hospital, Bao Ji, Shan Xi, China; 3 Long Chen, Department of Orthopedics, West China Hospital, Sichuan University, Chengdu, Sichuan, China; 4 Kun Peng, Department of Orthopedics, West China Hospital, Sichuan University, Chengdu, Sichuan, China; 5 Zhao Xu, Department of Orthopedics, West China Hospital, Sichuan University, Chengdu, Sichuan, China; 6 Dagang Zhang, Department of Orthopedics, West China Hospital, Sichuan University, Chengdu, Sichuan, China; 7 Zhou Xiang, Department of Orthopedics, West China Hospital, Sichuan University, Chengdu, Sichuan, China

**Keywords:** Bisphosphonate, Osteoporosis, Hip fracture

## Abstract

**Objective:**

This meta-analysis was conducted to investigate the efficacy of bisphosphonates for preventing recurrent hip fracture and reducing the mortality of elderly patient with hip fracture.

**Methods:**

The databases of Pubmed, Embase and Cochrane Library were searched. All randomized or prospective matched controlled trials that assessed the efficacy of bisphosphonate for elderly patients with hip fracture were included. Two researchers independently extracted data of the included articles and assessed the methodological quality which was assessed based on Jadad scoring system or Newcastle-Ottawa scale. The second hip fracture incidence, mortality and complications were compared between bisphosphonates and control groups.

**Results:**

Four studies including 3088 patients were included. Results showed that there were significant difference of second hip fracture (P<0.05) and mortality (P<0.05) between bisphosphonates group and control group. While no significant intergroup difference were observed for all complications.

**Conclusions:**

Bisphosphonates can prevent subsequent hip fracture, reduce the mortality, and does not increase the overall complications in elderly patients with hip fracture.

## INTRODUCTION

Hip fracture represents the most serious consequence of osteoporosis, which is related to increased mortality,^1^-^3^ diminished function of extremity and quality of life.^4^, ^5^ With the aging of the world population, the incidence of fragility fracture will significantly increase, and the annual number of hip fracture may double during the first half century.^6^, ^7^ However, prior hip fracture is a risk factor for subsequent fracture, which is associated with a more than 2.0-fold increased risk of subsequent fracture.^8^, ^9^ with the incidence range from 1% to 14.8%.^10^-^12^ In addition, the high rate of recurrent hip fracture, which is also related to high rate of bone loss,^13^ accompany greatly increase of mortality.^9^

Considering the grave consequences, measures must be initiated to prevent the second hip fracture occurrence. Unfortunately, osteoporosis is usually under-diagnosed and undertreated in elderly hip fracture patients.^14^ In recent years, bisphosphonates are widely used for treatment of osteoporosis, such as Alendronate, Risedronate and Zoledronicet al., as those agents have been approved to reduce the incidences of vertebral and hip fracture in elderly patients.^15^-^17^ However, whether the bisphosphonates can reduce the risk of second hip fracture is unknown. Although a few studies had focused on the issue, it is still controversial because of the small sample and inconformity of the outcome. In order to acquire a reliable conclusion, we compiled all available data from a few prospective or randomized researches and carried out a meta-analysis to assess the efficacy of biphosphonates on the subsequent hip fracture in the elderly patients with hip fracture.

## METHODS

### Systematic literature search

The databases of Pubmed, Embase and Cochrane Library were searched from inception to January 2015. The search strategy was based on combination of medical subject headings (MeSH) or keywords “bisphosphonates”, “osteoporosis”, “hip fracture”. Two researchers independently identified the titles and abstracts related to effect of bisphosphonates for elderly patient with hip fracture, and full text of all potentially relevant studies were obtained to identify whether it can be included. References of all included articles were also manually browsed to identify potentially relevant studies which were not searched in the databases.

### Inclusion and exclusion criteria

***Types of studies:*** Randomized control trial (RCT) and prospective non-randomized concurrent controlled trials were considered for this meta-analysis.

***Types of participants:*** Osteoporotic patients with age more than 50 years who had undergone hip fracture were included in this research. Patients with secondary osteoporosis were excluded.

***Types of interventions:*** Trials that compared oral bisphosphonate with placebo or blank control in older patients with hip fracture were included.

***Types of outcome measures:*** The primary outcomes were second hip fracture, mortality. The second hip fracture was related to the recurrent hip fracture. Secondary outcomes were the all complications, other complications. Other complications refer to the complications that excluded the mortality and the second hip fracture.

### Data extraction and quality assessment

Two researchers independently extracted data from the eligible articles, and performed the assessment of the methodological quality. Any disagreement was resolved by discussion to reach final consensus. If more than one paper with the same data were identified, only the one containing definitive data were included. Extracted data included demographics, methodology, details of intervention, and the interest outcomes (such as the second hip fracture cases, mortality and complications). If there were no exact records about the second fracture cases, an e-mail was sent to the authors in order to obtain the accurate cases. The quality of RCTs were assessed by the Jadad score,^18^ with a cumulative score >3 indicating high quality. While the quality of non-randomized trials were assessed by the Newcastle-Ottawa scale,^19^ a score ≥5 indicating high quality.

### Data analyses

All data analysis was conducted with the Review Manager 5.1 software. The weighted mean difference was measured for continuous variables, and relative risk (RR) was calculated for dichotomous outcomes. *P<*0.05 were considered statically significant, and the 95% confidence intervals (CIs) were reported. Statistical heterogeneity among researches was assessed by I-square (*I^2^*) and Chi-square (χ) test. If there was no statistical heterogeneity (*I^2^<*50% or χ^2^ test *P*≥0.1), a fixed effect model was adopted, or a random effect model was used. Funnel plots were not created because the included trials were only four.

## RESULTS

### Characteristics of studies

**Fig.1:**
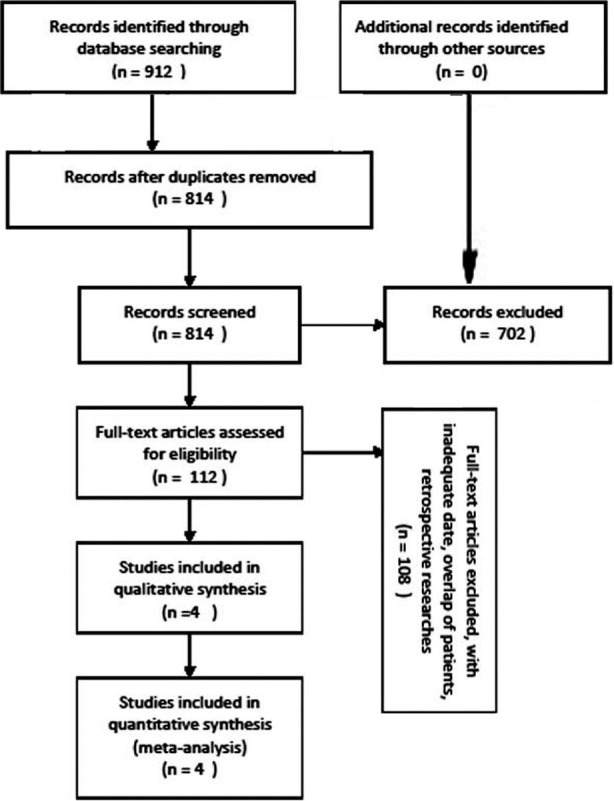
Flow diagram of articles identification.

A total of 912 studies were retrieved from the database after removing the duplication. After carefully screening of the titles and abstracts by two reviewers, another 702 papers were excluded because they were not found relevant. Then the two reviewers reviewed the full article, and 108 articles were excluded, leaving 4 articles^20^-^23^ which matched the inclusion criteria for data extraction and analysis. In the 4 researches, a total of 3088 patients over 50 years were enrolled in this meta-analysis (Fig.1). The baseline characteristics were comparable between the bisphosphonate group and control group, and the intervention contained *zoledronic acid, Alendronate, Risedronate, Etidronate* (Table-I). Outcomes of interest, such as second hip fracture, mortality, complications, are involved in those researches (Table-II). The outcomes of the quality assessment of the included researches were displayed in Table III and IV.

**Table-I:** Characteristics of included studies.

Included studies	Design	Samples		Age(years)		Men(%)		Intervention	Follow-up (month)

		Bisphosphonate	Control	Bisphosphonate	Control	Bisphosphonate	Control		
Lyles et al.[20]	RCT	1054	1057	74±9.48	74.6±9.86	23.3%	24.5%	Zoledronic acid	36
Beaupre et al.[22]	Prospective control	101	108	56% patients > 75 years	44%patients>75 years	27%	43%	Alendronate, Risedronate Etidronate	24
Cecilia et al.[21]	RCT	119	120	81±7	81±7	20.2%	21.6%	Alendronate	12
Osaki et al.[23]	Prospective control	173	356	80.2±7.9	81.9±8	0	0	Risedronate	36

**Table-II:** Outcomes of two groups in the included studies.

Included studies	Second hip fracture		BMD of TH		Death		Other complications	

	Bisphosphonate	Control	Bisphosphonate	Control	Bisphosphonate	Control	Bisphosphonate	control
Lyles et al.[20]	23/1054	33/1057	+2.6%	-1.0%	101/1054	141/1057	743/1054	678/1057
Beaupre et al.[22].	3/101	1/108	NA	NA	7/101	17/108	NA	NA
Cecilia et al.[21]	2/119	2/119	+0.79%	-1.78%	13/119	15/120	10/119	12/120
Osaki et al.[23]	5/173	32/356	NA	NA	NA	NA	32/173	55/356

**Table-III:** Quality assessment of the included RCTs in term of the Jadad scoring system.

Study	Randomized	Appropriate randomization	Appropriate double blinded	Description of withdrawals	Jadad score	Study quality
Lyles et al.[20]	Yes	Yes	Yes	Yes	5	High
Cecilia et al.[21]	Yes	Yes	No	Yes	3	Low

**Table-IV:** Quality assessment of the non-randomized controlled trials in term of the Newcastle-Ottawa scale.

Study	Selection star	Comparability star	Outcome star	Total star	Study quality
Beaupre et al.[22]	4	1	3	8	High
Osaki et al.[23]	4	1	3	8	High

### Second hip fracture

**Fig.2:**
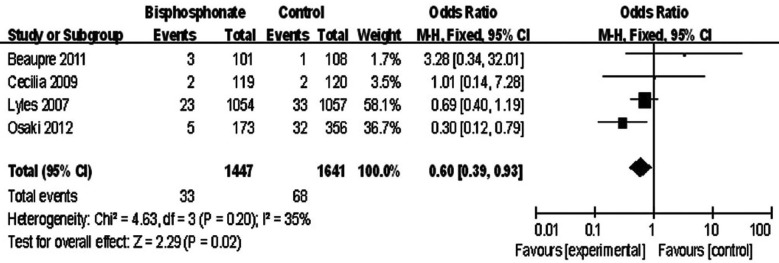
Comparison of second hip fracture between bisphosphonate group and control group.

The second hip fracture incidences were mainly compared in two researches.^20^, ^23^ Three researches showed the cases of second hip fracture of bisphosphonate group and control group. Another research^22^ revealed the total second hip fracture patients, and the specific numbers in each group was obtained by an e-mail from the authors. Overall, the four studies involved 3088 patients to compare the incidences of second hip fracture between the bisphosphonate group and control group. The heterogeneity test showed there was no statistical heterogeneity (χ^2^=4.63, df=3, *P*=0.20, *I*^2^=35%). Data pooled by the fixed effect model revealed significant difference of second hip fracture between bisphosphonate group and control group (mean difference: 0.60, 95% CI 0.39 to 0.93, *P*=0.02) (Fig.2).

### Mortality

**Fig.3:**
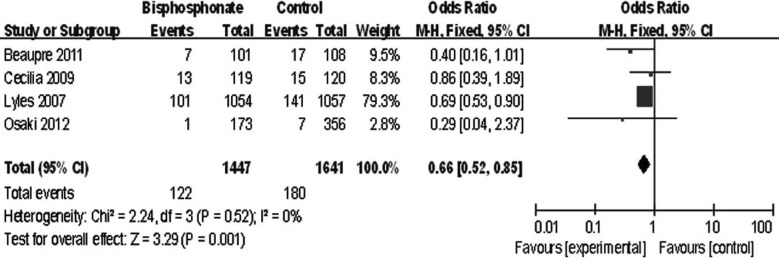
Comparison of mortality between bisphosphonate group and control group.

Two articles evaluated the mortality of bisphosphonate group and control group; although the other two studies did not directly assess the effect of bisphosphonate on the mortality, we can extract the cases of death of each group from the article. The heterogeneity test indicated there was no statistical heterogeneity (χ^2^=2.24, df=3, *P*=0.52, *I*^2^=0%). A fixed effect model was adopted and the analytic data showed that there was significant difference between bisphosphonate group and control group (mean difference: 0.66, 95% CI 0.52 to 0.85, *P*=0.001) (Fig.3).

### Other complications

**Fig.4:**
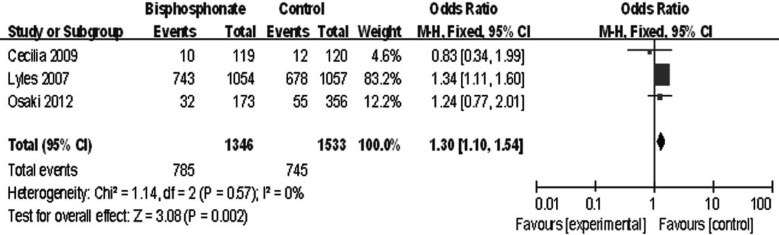
Comparison of other complications between bisphosphonate group and control group.

The other complications, which refer to the complications excluded the second hip fracture and death, included renal event, myalgia, influenza-like symptoms, headache, arthralgia, pyrexia, cardiovascular or cerebrovascular event, gastric symptoms, other sites fracture, etc. Three studies compared the other complications between two groups. The heterogeneity test indicated there was no statistical heterogeneity (χ^2^=1.14, df=2, *P*=0.57, *I*^2^=0%). The other complications were significant different between bisphosphonate group and control group (mean difference: 1.3, 95% CI 1.10 to 1.54, *P*=0.002) (Fig.4).

### All complications

**Fig.5:**
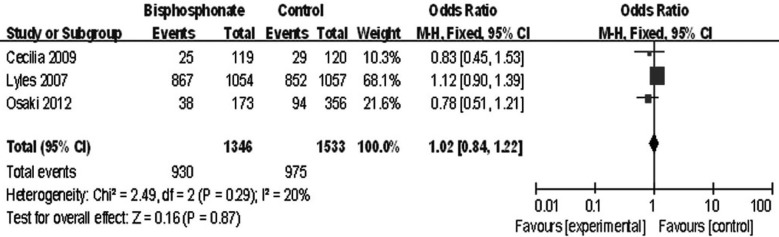
Comparison of all complications between bisphosphonate group and control group.

Data of all complications was extracted from the three studies and was analyzed. The heterogeneity test indicated there was no statistical heterogeneity (χ^2^=2.49, df=2, *P*=0.29, *I*^2^=20%). Data pooled by a fixed effect model revealed no statistical significant difference between the two groups (mean difference: 1.02, 95% CI 0.84 to 1.22, *P*=0.87) (Fig.5).

## DISCUSSION

Because of the subsequently increasing morbidity and mortality results from fragile hip fracture in elderly patient, measures must be carried out to prevent hip fracture. Anti-osteoporosis therapy—pharmacological treatment— is one of the most important methods which is now widely adopted presently. Bisphosphonates, such as alendronate, risedronate, zoledornic acid, recommended as first-line drug for primary prevention of osteoporotic fracture, are widely prescribed medicines for osteoporotic therapy, which were proved to be effective and can reduce the vertebral fracture, non-vertebral fracture and mortality.^15^, ^16^, ^24^-^26^ In addition, bisphosphonates also possessed a preventive effect on hip fracture in elderly osteoporotic patients.^27^ However, these researches mainly target the patients without hip fracture. As hip fracture in elderly osteoporotic patient indicated serious osteoporosis, and the prior fracture also is a risk factor for new fracture, whether the bisphosphonate can also hold the efficacy for preventing second hip fracture in elderly patients with hip fracture is under discussion.

The research focusing on the efficacy of preventing second hip fracture was very few. Although a few retrospective studies showed that adherent use of bisphosphonate could significantly reduce the risk of second hip fracture.^12^, ^28^ However, retrospective studies may affect the validity, and randomized control trial or prospective matched studies also needed to certify the efficacy of bisphosphonate on second hip fracture protection. In this meta-analysis, four articles were included, which contain two randomized control trials and two prospective studies. In general, only randomized control trial can be included in meta-analysis, but the researches that focused on the elderly patients with hip fracture and put the second hip fracture or mortality as the first outcome were so few that we included the other two prospective studies, in order to avoid losing the valid data. In the four researches, the baseline characteristics (such as age, BMD) between the bisphosphonate and control group were relatively comparable, which can reduce the selection bias. Finally, a total of 3088 patients were included in this analysis, and the outcomes revealed that there was significant difference of second hip fracture (mean difference: 0.60, 95% CI 0.39 to 0.93) and mortality (mean difference: 0.66, 95% CI 0.52 to 0.85) between bisphosphonate group and control group. The reduction of second hip fracture may be partly ascribed to the improved BMD produced by bisphosphonate, as a few researches showed than bisphosphonate can increase the BMD in patients with low trauma fracture.^20^, ^29^ The all complications were comparable between bisphosphonate group and control group (mean difference: 1.02, 95% CI 0.84 to 1.22), while the other complications (excluding the second hip fracture and mortality) were more common in the bisphosphonate group ((mean difference: 1.3, 95% CI 1.10 to 1.54). The results showed that constituent ratio of complications may be transformed by bisphosphonate—the rate of serious adverse events (such as death, second hip fracture) was reduced and some mild symptoms (such as gastric symptoms, headache, myalgia, influenza-like symptoms) increased.

The compliance with bisphosphonate also influence the efficacy of pharmacotherapy, and some researches had confirmed that the refracture rate for patients with >80% compliance was significantly lower than that <80% compliance.^12^, ^28^, ^30^ In this meta-analysis, the compliance of patients in the four included researches was higher than 80%. The decreased mortality can be attributed to several reasons. First, the reduction in the risk of death may be partly related to the reduction of second hip fracture, as subsequent hip fracture were significantly associated with excess mortality. Second, bisphosphonate could provide protective effect against cardiovascular events, and patients with bisphosphonate therapy had a lower risk of acute myocardial infarction, which was proved by Kang’s research.^31^ In addition, Colon-Emeric et al also reported that patients treated with zoledronic acid were less likely to die from cardiac arrhythmias than control group.^32^ Last, bisphosphonate may have effect on immunomodulatory effect, affecting both dendritic cells and T cells.^33^

### Limitations

First, the drugs, doses, frequency, prescription time in each research were not perfectly same, which may influence the outcomes of interest, although the drugs all belong to bisphosphonate and the doses were the recommended dose. Second, the included studies were still relatively few—only four researches. Last, because the researches of bisphosphonate for elderly patients with hip fracture are so few that two prospective matched cohort researches were also included, which may result in selection bias in this meta-analysis.

In conclusion, this meta-analysis revealed that bisphosphonates can prevent subsequent hip fracture, reduce the mortality, and does not increase the overall complications in elderly patient with hip fracture. On the basis of this meta-analysis, bisphosphonate therapy for elderly patients with hip fracture was supported.
